# The Role of Thermal Accumulation on the Fabrication of Diffraction Gratings in Ophthalmic PHEMA by Ultrashort Laser Direct Writing

**DOI:** 10.3390/polym12122965

**Published:** 2020-12-11

**Authors:** Daniel Sola, Javier R. Vázquez de Aldana, Pablo Artal

**Affiliations:** 1 Institut für Fertigungstechnik, Technische Universität Dresden, 01069 Dresden, Germany; 2 Laboratorio de Óptica, Centro de Investigación en Óptica y Nanofísica, Campus Espinardo, Universidad de Murcia, 30100 Murcia, Spain; pablo@um.es; 3 Aplicaciones del Láser y Fotónica, University of Salamanca, 37008 Salamanca, Spain; jrval@usal.es

**Keywords:** ultrafast laser inscription, laser materials processing, polymers, diffraction gratings, ophthalmic material

## Abstract

The fabrication of diffraction gratings by ultrashort direct laser writing in poly-hydroxyethyl-methacrylate (PHEMA) polymers used as soft contact lenses is reported. Diffraction gratings were inscribed by focusing laser radiation 100 µm underneath the surface of the samples. Low- and high-repetition rate Ti:sapphire lasers with 120 fs pulsewidth working at 1 kHz and 80 MHz respectively were used to assess the role of thermal accumulation on microstructural and optical characteristics. Periodic patterns were produced for different values of repetition rate, pulse energy, laser wavelength, distance between tracks, and scanning speed. Compositional and structural modifications of the processed areas were studied by micro-Raman spectroscopy showing that under certain parameters, thermal accumulation may result in local densification. Far-field diffraction patterns were recorded for the produced gratings to assess the refractive index change induced in the processed areas.

## 1. Introduction

During the last decades, polymers have entered in almost all industrial, technological, and biotechnological applications due to their excellent bulk physical and chemical properties such as low surface energy, hydrophobicity, and high electrical resistance [1,2]. Polymers are the largest class of materials used for biomedical applications because manufacturing is easy and reliable, highly efficient, and energy saving. In biomedicine, they have been applied as orthopedic, dental, hard, and soft tissue replacements, cardiovascular devices, drug delivery, and as both contact and intraocular lenses [1,2,3,4,5,6,7,8]. In particular, poly-hydroxyethyl-methacrylate (PHEMA) and silicone hydrogels have been commonly used as soft contact and intraocular lenses because of their oxygen permeability, biocompatibility, biostability, durability, flexibility, and transparency from the UV to the NIR spectral region [1,4,9,10].

Ultrashort laser direct writing has been widely used for structuring polymers, crystals, and glasses to functionalize selectively the surface, to create 2D/3D micro/nanostructures, and to produce passive and active photonic devices [11,12,13,14,15,16,17,18,19,20,21,22,23,24,25,26,27,28,29,30,31,32,33,34,35]. The absorption process of ultrashort laser pulses results in high local spatial and temporal electronic and vibrational excitation densities. In addition, laser pulse duration, which is short compared to the relevant relaxation processes, gives rise to laser-induced nonlinear processes in the focal volume, such as multiphoton absorption, inducing avalanche ionization in a very short time. These phenomena lead to localized micro or sub-micrometric lattice damage, permanent weak local refractive index variation, the formation of nano-voids, crystallization processes, or chemical transformations [11,12]. Short and ultrashort laser radiation has been recently used to modify the refractive power of polymers for ophthalmic applications by inscribing diffractive optical elements on the surface or inside the material [15,18,19,22,36,37,38,39,40]. Nevertheless, the role that thermal accumulation plays on the optical properties of diffraction gratings produced by ultrashort direct laser writing for ophthalmic applications has not conclusively been solved.

We report here on the fabrication of diffractive gratings in PHEMA polymers used as soft contact lenses by using low- and high-repetition rate ultrashort laser pulses. This type of structure has been recently proposed to be used for refractive correction [15,18,36,37]. Periodic linear patterns were inscribed underneath the surface sample modifying laser pulse energy, laser wavelength, repetition rate, scanning speed, and inter-line spacing. This variation of working parameters allows evaluating the role of thermal accumulation in the characteristics of the laser-written diffraction gratings and to control the damage induced in the sample. This issue is of great importance for ophthalmic applications in soft contact lenses, ex vivo, and in vivo refractive correction, which is required to achieve the highest refractive index modification in the shortest processing time, minimizing the damage induced in the material. Optical and phase contrast microscopy, confocal micro-Raman spectroscopy, and recording of far-field patterns were used to characterize processed samples.

## 2. Experimental System

### 2.1. Laser Setup

**Figure 1:**
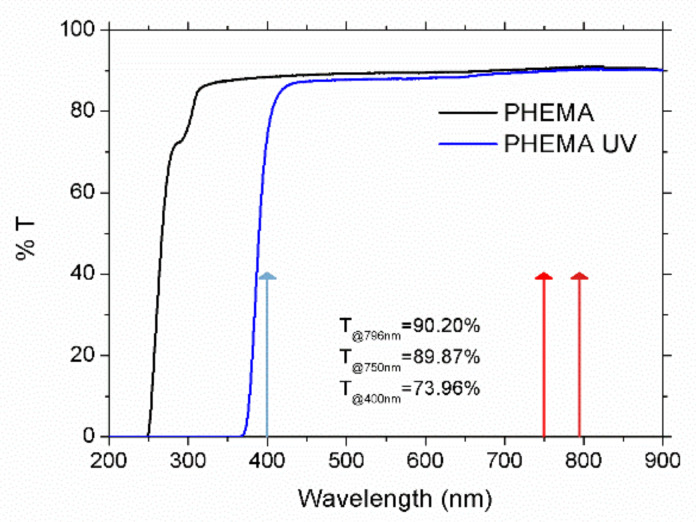
Optical transmission spectra of poly-hydroxyethyl-methacrylate (PHEMA) and PHEMA-UV samples. Arrows point out the laser wavelengths used to process the samples, and the optical transmittance at these wavelengths is also indicated.

Diffraction gratings were fabricated using low- and high-repetition rate laser sources. These two laser systems consist of a regenerative amplifier Ti:sapphire laser delivering 120 fs pulses at a central wavelength of 796 nm at a repetition rate of 1 kHz (Spitfire, Spectra Physics, Santa Clara, CA, USA), and a Ti:sapphire oscillator delivering 120 fs laser pulses at a repetition rate of 80 MHz (MaiTai HP, Spectra Physics, Santa Clara, CA, USA). This latter system was tuned at 750 nm to process the samples in the visible spectral region. In addition, to study the influence of the laser wavelength, a second harmonic generator (SHG) (Inspire Blue, Spectra Physics, Santa Clara, CA, USA) was used in the oscillator laser source to deliver laser pulses at 400 nm under the same conditions of pulse duration and repetition rate. For both laser sources, the beam was focused 100 µm underneath the surface by a 20× long working distance infinity corrected microscope objective, with 0.42 numerical aperture. The sample, placed in a 3D motorized stage, was scanned to produce parallel lines with lateral separation of 10, 20, and 40 µm. Pulse energy was set at 0.09 and 0.12 µJ for the regenerative amplifier and from 0.19 to 3 nJ for the laser oscillator. These values were selected after previous experiments to be above the modification threshold and not to induce an excessive damage on the samples. As the substrate, 1 mm thick poly-hydroxyethyl-methacrylate (PHEMA) polymer samples employed as soft contact lens (Contamac Ltd., Saffron Walden, UK) were used. This polymer, when used for ophthalmic applications, incorporates UV filters to shift the transmission cut-off wavelength at 375 nm, as shown in Figure 1. Optical transmittance at the laser wavelengths used to process the samples was 90.20%, 89.87%, and 73.96% at 796, 750, and 400 nm, respectively. The processing parameters are summarized in Table 1.

### 2.2. Characterization Techniques

Optical transmission spectra were obtained by means of a spectrophotometer (U-3400, Hitachi, Abingdon, UK). Bright field and phase contrast images were taken with a microscope (B-800PH, Optika, Ponteranica, Italy). Microstructural and chemical modifications in laser-treated areas were assessed by using confocal micro-Raman spectroscopy. The system consists of a confocal microscope and a spectrometer (SR303i-B, Andor, Belfast, Northern Ireland) equipped with a thermoelectric-cooled CCD detector (Newton 920, Andor). The excitation laser source is delivered to the confocal optical microscope and focused onto the sample by using a microscope objective. The microscope is provided with microscope objectives of 10× (0.25 NA), 20× (0.4 NA) and 60× (0.85 NA). A backscattered Raman signal is collected by the selected microscope objective and delivered to the spectrograph and the CCD detector. Software provided by the manufacturer (Andor Solis) allows plotting and managing the collected signal. Specifically, in this work, characterization of the samples was performed by placing the samples in a cross-section view. As the excitation source, a continuous wave 532 nm laser was used. The backscattered signal was collected through the 60× (0.85 NA) microscope objective lens, which provided a laser beam diameter of 1.5 µm at the focal plane. To avoid significant local heating of the sample, the output power of the laser was kept below 20 mW. Finally, diffractive modes were characterized by illuminating the diffraction gratings with a continuous 3 mW He-Ne laser emitting at 632.8 nm.

## 3. Results and Discussion

### 3.1. Laser Processing

#### 3.1.1. Thermal Accumulation by Ultrashort Laser Pulses: Background

In materials laser processing, one of the most critical parameters to take into account is the repetition rate, which is also called the frequency, because excitation and relaxation phenomena at the focal spot volume are affected by the time between subsequent laser pulses. High repetition rates lead to thermal accumulation in the laser focal volume, resulting in a great increase of the local temperature followed by a fast annealing process that may induce substantial changes in the microstructural properties of the laser processed areas [32,33,34,35]. The repetition rate at which the cross over from non-thermal to the thermal regime is produced is called the critical frequency and can be estimated as [20]:

(1) $$f_{cr}=\frac{D_{th}}{d_{laser}^{2}},$$

where *D_th_* is the thermal diffusivity and *d_laser_* is the laser beam diameter at the focal plane. The thermal diffusivity of PHEMA polymers ranges 10^−7^ m^2^/s [41]. In the case of a diffraction-limited focusing by a numerical aperture lens, the minimum waist diameter is given by *d*_0_ = 2⸱1.22⸱*λ*/*NA* [11] giving rise to beam diameters of 4.6, 4.3, and 2.3 µm for 796, 750, and 400 nm, respectively. Hence, the critical frequency ranges are 5 kHz for 796 nm and 750 nm, and 18 kHz for 400 nm. Accordingly, laser irradiated micron-sized regions will cool in a time of *t*~*1*/*f_cr_* microseconds so that the laser effect of multiple laser pulses focused into the same point will accumulate if the period between the pulses is shorter than this cooling time. To distinguish whether laser processing is carried out in a non-thermal or thermal regime, a normalized frequency parameter, *f_n_* = *f_laser_*/*f_cr_*, is commonly utilized. Therefore, if *f_n_* < 1, laser processing takes place in a non-thermal regime, whereas if *f_n_* ≥ 1, laser processing is carried out in a thermal regime [20].

#### 3.1.2. Laser Processing in Non-Thermal Regime

**Figure 2:**
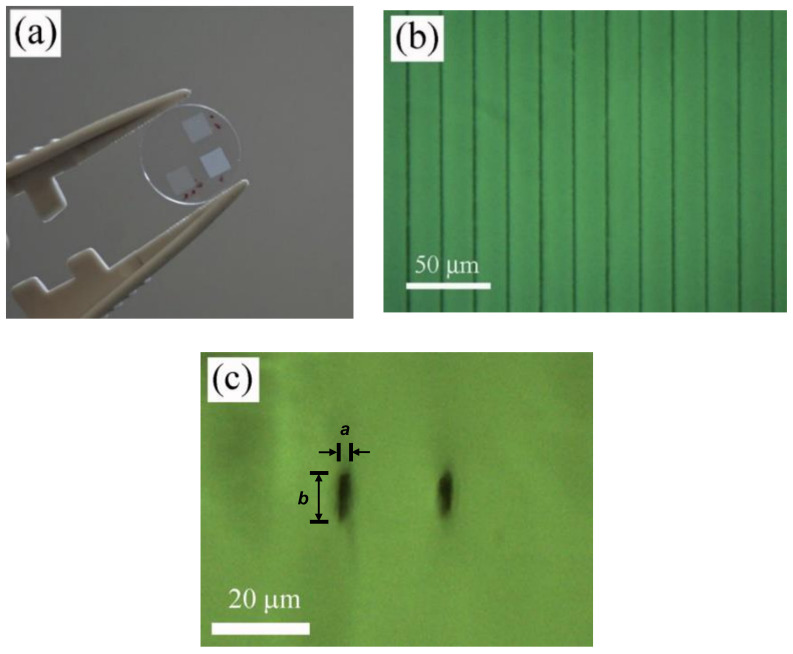
General view of the gratings inscribed with the laser amplifier at 796 nm, 0.50 mm/s, and inter-line spacing of 10, 20, and 40 µm using laser pulse energy of 0.09 µJ, (**a**), and top-view and cross-section-view microscope images in bright field mode of the sample processed with 20 µm inter-line spacing, 0.09 µJ laser pulse energy, and 0.50 mm/s scanning speed, (**b**,**c**).

In the case of the Ti:sapphire amplifier, the normalized frequency was *f_n_*~0.2. Accordingly, the cooling process takes place quickly so that thermal accumulation can be considered insignificant and the heat-affected zone outside the focal volume is almost negligible. Although the linear absorption at the laser wavelength used to inscribe the linear tracks was very low, the laser intensity utilized to process the polymer sample was high enough to induce nonlinear absorption processes, resulting in permanent changes in the structure of the polymer. In particular, linear gratings were written 100 µm underneath the surface of the sample by translating the samples at 0.25, 0.50, and 1 mm/s using a laser pulse energy of 0.09 µJ. To compare the effect of the laser pulse energy, the grating processed at 1 mm/s was also written with a pulse energy of 0.12 µJ. As an example, Figure 2a shows a general view of the gratings inscribed at 0.50 mm/s and inter-line spacing of 10, 20, and 40 µm using a laser pulse energy of 0.09 µJ, and Figure 2b,c show top-view and cross-section-view microscope images in bright field mode of the sample processed with 20 µm inter-line spacing, 0.09 µJ laser pulse energy, and 0.50 mm/s scanning speed, respectively. As can be observed, tracks written at a low repetition rate and high pulse energy induced strong nonlinear absorption, leading to an asymmetric filament shape. The morphology of the inscribed tracks was measured with an optical microscope. The width of these tracks, *a*, was found to be around 4 µm, whereas the thickness, *b*, varied depending on the processing parameters. In particular, the grating thickness resulted in 9, 8, and 7 µm at 0.25, 0.50, and 1 mm/s, respectively, for 0.09 µJ laser pulse energy, and 9 µm for 0.12 µJ and 1 mm/s. 

#### 3.1.3. Laser Processing in Thermal Regime

High-repetition-rate low-pulse energy laser sources, properly combined with high numerical aperture focusing objectives, have been used to induce nonlinear absorption processes in the thermal regime to create permanent structures in transparent materials [32,33,34,35,36,38,39,40]. In this work, normalized frequencies for the Ti:sapphire oscillator were *f_n_*~16 × 10^3^ and 4.7 × 10^3^ at 750 nm and 400 nm, respectively. Accounting that they were well above the critical frequency, laser processing was carried out in a thermal regime. 

**Figure 3:**
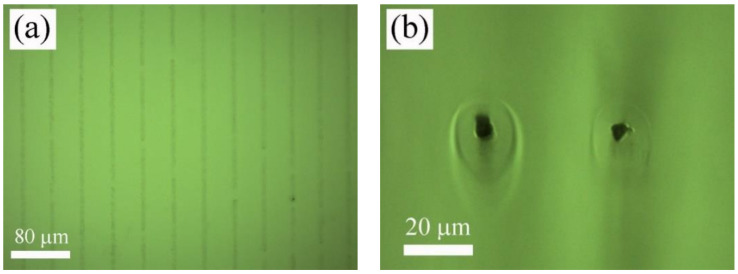
Top-view (**a**) and cross-section-view (**b**) microscope images in bright field mode of the sample processed with the laser oscillator at 750 nm, with 40 µm inter-line spacing, 1.5 nJ pulse energy, and 0.050 mm/s.

In the first place, departing from 800 nm, the laser wavelength was tuned until it was possible to induce permanent marks inside the material. This effect was firstly achieved at a laser wavelength of 750 nm, which coincides with double the cut-off wavelength of the transmission spectrum of PHEMA-UV polymer, which was placed at 375 nm, as shown in Figure 1. At this wavelength, it was possible to create these structures only at low scanning speed. In particular, diffraction gratings were inscribed 100 µm underneath the surface of the sample by using 1.5 nJ and 3 nJ laser pulse energies and 0.025, 0.050, and 0.100 mm/s scanning speed. Therefore, at this wavelength, the structuring process was highly inefficient, requiring processing rates one order of magnitude lower than those used with the Ti:sapphire amplifier. As an example, Figure 3 shows top-view (a) and cross-section-view (b) microscope images in the bright field mode of the sample processed with 40 µm inter-line spacing, 1.5 nJ and 0.050 mm/s. A cross-section view revealed that the inscribed tracks were circular in shape. In addition, a ring-shaped area around the structure was observed, suggesting regions with different refractive index. As shown in Figure 3b, the radius of this ring-shaped area ranged 9 µm from the center of the track. Since this area might induce non-desired effects for the purposes of the present work, diffraction gratings inscribed with this laser wavelength were fabricated with inter-line spacings of 20 and 40 µm. In this case, the radius of the track was 6 µm and 5 µm at 0.050 mm/s and 0.100 mm/s, respectively, for 1.5 nJ laser pulse energy, and 7 µm for 3 nJ and 0.100 mm/s. This type of structure has previously been reported in the laser writing of transparent materials with laser sources at low pulse energy and high repetition rates. They are produced by accumulative heating of the material followed by non-uniform resolidification [11,12,32,33,34,35].

**Figure 4:**
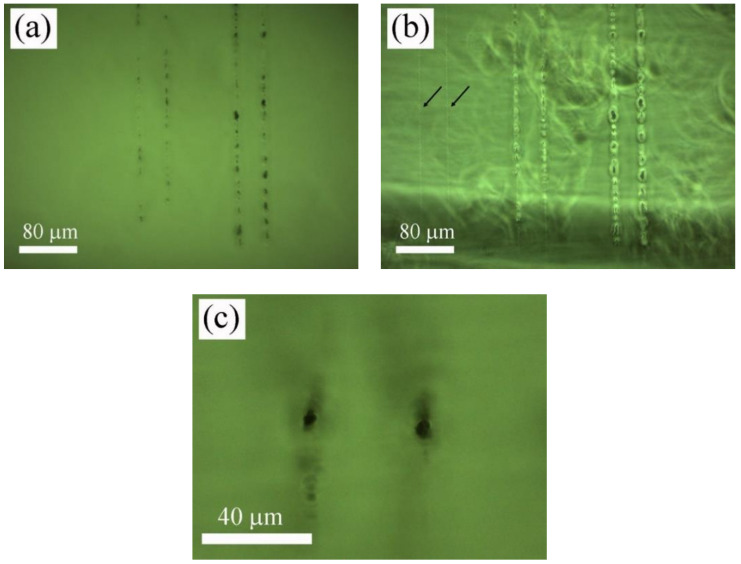
Top-view microscope images in bright field and contrast phase mode, (**a**,**b**) respectively, of the tracks written inside the sample with the laser oscillator at 400 nm as a function of the laser intensity by translating the sample at 0.25 mm/s. Arrows in (**b**) points out tracks written at 0.19 nJ. (**c**) shows a cross-section-view microscope image in bright field mode of the tracks structured at 0.38 nJ and 0.25 mm/s.

Next, samples were processed at a high repetition rate, tuning the laser wavelength at 400 nm by means of a second harmonic generator (SHG). At this wavelength, it was possible to induce permanent structural changes inside the polymer samples at the same scanning speeds used in the case of the Ti:sapphire amplifier, i.e., 0.25, 0.50, and 1 mm/s. In particular, linear gratings were inscribed with 0.19, 0.38, and 0.75 nJ laser pulse energies. Figure 4 shows top-view microscope images in bright field and contrast phase mode, Figure 4a,b, respectively, of the tracks written inside the sample as a function of the laser intensity by translating the sample at 0.25 mm/s. Arrows in Figure 4b point out tracks written at 0.19 nJ. Figure 4c shows a cross-section-view microscope image in bright field mode of the tracks structured at 0.38 nJ and 0.25 mm/s. It can be observed that at high laser pulse energy conditions, laser interaction with the polymer sample resulted in inhomogeneous heat accumulation combining areas of low and high damage and producing local degradation in the sample. However, for the case of the lowest pulse energy utilized to structure the sample, tracks were found to be homogeneous without apparent degradation and a size close to the laser beam diameter. It is worth noting that these marks were only visible in the phase contrast mode. It has been reported that this type of laser-induced structure is produced below the damage threshold [15,18,36,37]. Concerning the shape of the structured tracks, as in the previous case, they were circular in shape. Nevertheless, unlike the tracks written at 750 nm, the ring around the structured track was not observed. In this case, the radius of the tracks were found to be 4.5 µm, 4 µm, and 3.5 µm at 0.25 mm/s, 0.50 mm/s, and 1 mm/s, respectively, for 0.19 nJ laser pulse energy, and 7 µm for 0.38 nJ and 1 mm/s.

Finally, the temperature reached on the irradiated areas was estimated according to the one-dimensional heat conduction equation [42]:

(2) $$\frac{\partial T}{\partial t}=D_{th}\frac{\partial T}{\partial x^{2}}\;,$$

where *D_th_* is the thermal diffusivity. The solution for a single laser pulse is given by:

(3) $$T_{m}=\frac{2}{\sqrt{\pi }}\frac{I_{a}{\left(D_{th}t_{p}\right)}^{1/2}}{k},$$

where *I_a_* is the laser intensity, *t_p_* is the pulse duration, and *k* is the heat conduction coefficient, the value of which is 1.74 × 10^−3^ W/cm⸱K for PHEMA polymer. In the case of a succession of laser pulses, average temperature is provided by:

(4) $$\bar{T_{n}}≅2T_{m}{\left(t_{p}R\right)}^{1/2},$$

where *R* is the repetition rate. Estimated average temperatures were found to be 700 K and 934 K in the non-thermal regime at 796 nm laser wavelength, and 0.09 µJ and 0.12 µJ pulse energy, respectively. In the thermal regime, the average temperature took values of 3779 K and 7557 K at 750 nm and 1.5 nJ and 3 nJ pulse energy, respectively, and 1673 K and 3346 K at 400 nm and 0.19 nJ and 0.38 nJ, respectively.

### 3.2. Microstructural Characterization

**Figure 5:**
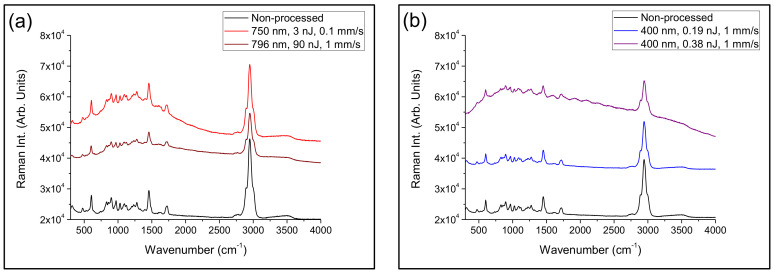
Raman spectra in the wavenumber region 300−4000 cm^−1^ of non-processed areas and laser-structured regions with the laser amplifier at 796 nm and with the oscillator at 750 nm, (**a**), and with the laser oscillator at 400 nm at high and low laser pulse energy, (**b**).

A laser-induced modification of both polymer structure and chemical composition in processed areas were investigated by confocal µ-Raman spectroscopy. Characterization of the samples was performed by placing the samples in a cross-section view, as shown in Figure 2c, Figure 3b, and Figure 4c. Accounting that the width of the processed areas ranged from 3.5 to 7 µm, the diameter of the laser excitation source at the focal plane, 1.5 µm, allows acquiring the signal with suitable precision and accuracy. Figure 5 shows Raman spectra in the wavenumber region 300–4000 cm^−1^ of non-processed areas and laser-structured regions with the laser amplifier at 796 nm and with the oscillator at 750 nm, Figure 5a, and with the laser oscillator at 400 nm at high and low pulse energy, as shown in Figure 5b. Raman spectra showed sharp peaks and broad bands, which agree with those previously reported in the literature [43,44,45]. These bands and peaks were assigned as follows: 475 cm^−1^, deformation mode; 604 cm^−1^, *ν_s_*CCO; 831 cm^−1^, *ν_s_*COC; 899 cm^−1^, *ν_s_*COC(H); 970 cm^−1^, *ρ*CH_3_; 1029 cm^−1^, *ν*CC; 1094 cm^−1^, *ν_as_*OCH_2_C, *ρ*CH_3_, and *ρ*CH_2_; 1204 cm^−1^, *τ*CH_2_ and *ω*CH_2_; 1279 cm^−1^, *τ*CH_2_ and *ω*CH_2_; 1458 cm^−1^, *δ*CH_2_ and *δ*CH_3_; 1724 cm^−1^, *ν*C=O; and 2949 cm^−1^, *ν_as_*CH_2_. 

Raman spectra of areas structured with the laser amplifier at 796 nm showed strong intensity diminution, mainly in the components placed at 604, 1458, 1724, and 2949 cm^−1^, which results from the damage induced by the ultra-intense laser radiation. In addition, a small increase in the background in the wavenumber range 500–2000 cm^−1^ was observed. Concerning samples processed with the laser oscillator at 750 nm, Raman spectra presented a strong fluorescence background increase associated to the thermal decomposition of organic molecules [45]. In addition, peak intensity variation was not as significant as in the previous case. Regarding samples processed with the laser oscillator at 400 nm, at high laser pulse energy, both a strong fluorescence background increase and peak intensity diminution were observed so that laser radiation induced photo-thermal decomposition and structure degradation. Nevertheless, at low pulse energy, there were no significant changes in the Raman spectra and hence, the polymer structure remained almost unaltered after laser irradiation. In this case, laser radiation induced additional cross-linking and local densification [36,37]. 

### 3.3. Optical Characterization

**Figure 6:**
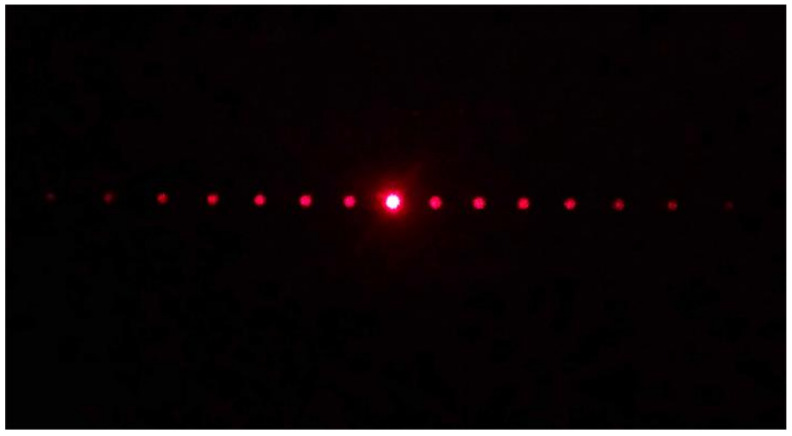
Far-field diffraction image of the output beam transmitted through the periodic pattern processed with the laser amplifier at 796 nm, with 20 µm inter-line spacing, 0.09 µJ laser pulse energy, and 0.25 mm/s.

The characterization of periodic patterns was carried out under illumination of a continuous-wave He-Ne laser with emission at 632.8 nm for which the angle of incidence was set orthogonal to the samples. All structured samples showed diffraction patterns with diffraction angles in agreement with the diffraction equation *mλ* = *Λsinθ*, where *m* is the diffraction order, *λ* the laser wavelength, and *Λ* is the grating period. As an example, Figure 6 shows a far-field diffraction image of the output beam transmitted through the periodic pattern processed with the laser amplifier, with 20 µm inter-line spacing, 0.09 µJ laser pulse energy, and 0.25 mm/s. 

**Figure 7:**
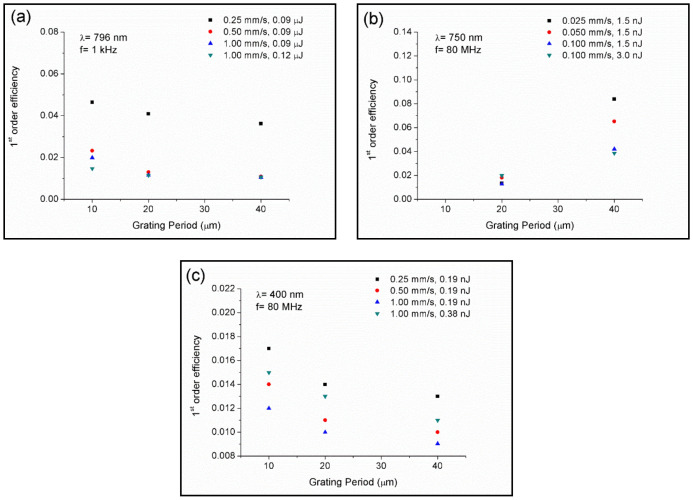
First-order efficiency of the samples processed with the laser amplifier at 796 nm (**a**), laser oscillator at 750 nm (**b**), and laser oscillator at 400 nm (**c**).

Next, zero- and first-order intensities were measured by using a power meter to determine the 1st-order efficiency. Figure 7a–c show the 1st-order efficiency of the samples processed with the laser amplifier at 796 nm, laser oscillator at 750 nm, and laser oscillator at 400 nm, respectively. It was observed that samples processed at 796 and 400 nm presented similar behavior. First-order efficiency decreased as the inter-line spacing was increased, whereas for a given inter-line spacing, it was increased by decreasing the scanning speed. The effect of the increase in laser pulse energy resulted in opposite behavior. For the case of the laser amplifier, 1st-order efficiency was decreased, whereas for the case of the oscillator at 400 nm, it was increased. It is worth noting the behavior observed in samples processed with the laser oscillator at 750 nm. The 1st-order efficiency increased by both increasing the inter-line spacing or decreasing the scanning speed. Changes in the laser pulse energy did not provide significant variations. This behavior might be associated with the ring-shaped zone observed around the inscribed tracks, as shown in Figure 3b.

Refractive index modification, Δ*n*, was assessed under assumption of a Raman–Nath diffraction grating [46,47,48]:

(5) $$\Delta n=\frac{\lambda cos\theta tanh^{-1}\left(\sqrt{\eta }\right)}{\pi b},$$

where *λ* is the laser wavelength used to evaluate the gratings, 633 nm, *θ* is the angle of incidence from the normal in the media, which according to our experimental conditions was set at 0°, *η* is the 1st-order efficiency, I_1_/I_0_, and *b* is the grating thickness, which was measured with an optical microscope in a cross-section view. 

Samples processed in a non-thermal regime, with the laser amplifier at 796 nm, showed a refractive index change ranging 2.99 × 10^−3^ and 6.30 × 10^−3^. In the thermal regime, the refractive index variation ranged between 3.36 × 10^−3^ and 8.78 × 10^−3^ in samples processed with the laser oscillator at 750 nm, and between 3.00 × 10^−3^ and 7.55 × 10^−3^ in samples processed at 400 nm. Values obtained in both the non-thermal and thermal regime were similar to those reported by using the ultrafast laser writing technique in acrylate and silicone polymers [47,48,49,50].

## 4. Conclusions

Ultrashort direct laser writing was applied to produce linear periodic patterns underneath the surface of poly-hydroxyethyl-methacrylate (PHEMA) polymers employed as soft contact lenses using femtosecond laser pulses in non-thermal and thermal regimes. Laser-inscribed periodic patterns were assessed as a function of inter-line spacing, scanning speed, and pulse energy. The shape of filaments written in the non-thermal regime was found to be asymmetric, whereas in the thermal regime, they were circular in shape. It is worth noting that when processed at 750 nm, a ring around the filament structure was observed, suggesting regions with different refractive indexes. Compositional and microstructural characterization carried out by micro-Raman spectroscopy showed that in the non-thermal regime, laser radiation induced damage on the polymer structure. Thermal accumulation at a high repetition rate and high irradiance lead to photo-thermal damage and decomposition. Nevertheless, in a thermal regime and at low laser pulse energy, it was possible to inscribe tracks below the damage threshold for which the polymer structure remained almost unaltered after laser irradiation and which was visible only under phase contrast microscopy. Optical characterization showed that all periodic patterns presented diffraction orders under 632.8 nm cw-He-Ne laser illumination. First-order efficiency presented a similar behavior in the non-thermal regime at 796 nm and the thermal regime at 400 nm, whereas it was found to be opposite at a high repetition rate at 750 nm. Refractive index changes induced by laser radiation ranged from 2.99 × 10^−3^ to 8.78 × 10^−3^. These results determine that a laser oscillator at 400 nm is the optimal laser system for fabricating diffraction gratings for ophthalmic purposes. This system allows producing these optical devices at a high processing rate, without inducing damage to the material, achieving a high refractive index change. 

## Figures and Tables

**Table 1:** Processing parameters utilized to fabricate the diffraction gratings.

Laser Source	Wavelength (nm)	Repetition Rate (kHz)	Scanning Speed (mm/s)	Inter-Line Spacing (µm)	Pulse Energy (nJ)
Amplifier	796	1	0.25, 0.5, 1	10, 20, 40	90, 120
Oscillator	750	80 × 10^3^	0.025, 0.05, 0.1	20, 40	1.5, 3.0
Oscillator	400	80 × 10^3^	0.25, 0.5, 1	10, 20, 40	0.19, 0.38, 0.75

## References

[1] Rubinstein M., Colby R.H. (2003). Polymer Physics.

[2] Schnabel W. (2007). Polymers and Light.

[3] Hussain F., Hojjati M., Okamoto M., Gorga R.E. (2006). Polymer-matrix Nanocomposites, processing, manufacturing, and application: An overview. J. Compos. Mater..

[4] Scholz C. (2017). Polymers for Biomedicine: Synthesis, Characterization and Applications.

[5] Deligkaris K., Tadele T.S., Olthuis W., Berg A. (2010). Hydrogel devices for biomedical applications. Sens. Actuators B.

[6] Hunter A.C., Moghimi M.S. (2017). Smart polymers in drug delivery: A biological perspective. Polym. Chem..

[7] Calo E., Khutoryanskiy V.V. (2015). Biomedical applications of hydrogels: A review of patents and commercial products. Eur. Polym. J..

[8] Maulvi F.A., Soni T.G., Sha D.O. (2016). A review on therapeutic contact lenses for ocular drug delivery. Drug Deliv..

[9] Allen N.S. (2010). Photochemistry and Photophysics of Polymeric Materials.

[10] Belluchi R. (2013). An introduction to intraocular lenses: Material, optics, haptics, design and aberration. Cataract.

[11] Misawa H., Juodkazis S. (2006). 3D Laser Microfabrication.

[12] Osellame R., Cerullo G., Ramponi R. (2012). Femtosecond Laser Micromachining, Photonic and Microfluidic Devices in Transparent Materials.

[13] Davis K.M., Miura K., Sugimoto N., Hirao K. (1996). Writing waveguides in glass with a femtosecond laser. Opt. Lett..

[14] Nolte S., Will M., Burghoff J., Tuennermann A. (2003). Femtosecond waveguide writing: A new avenue to three-dimensional integrated optics. Appl. Phys. A.

[15] Ding L., Blackwell R., Künzler J.F., Knox W.H. (2006). Large refractive index change in silicone-based and non-silicone-based hydrogel polymers induced by femtosecond laser micro-machining. Opt. Express.

[16] Gamaly E.G., Juodkazis S., Misawa H., Luther-Davies B., Rode A.V., Hallo L., Nicolai P., Tikhonchuk V.T. (2008). Formation of nano-voids in transparent dielectrics by femtosecond laser. Curr. Appl. Phys..

[17] Ding L., Blackwell R.I., Künzler J.F., Knox W.H. (2008). Femtosecond laser micromachining of waveguides in silicone-based hydrogel polymers. Appl. Opt..

[18] Ding L., Jani D., Linhardt J., Künzler J.F., Pawar S., Labenski G., Smith T., Knox W.H. (2008). Large enhancement of femtosecond laser micromachining speed in dye-doped hydrogel polymers. Opt. Express.

[19] Ding L., Jani D., Linhardt J., Künzler J.F., Pawar S., Labenski G., Smith T., Knox W.H. (2009). Optimization of femtosecond laser micromachining in hydrogel polymers. J. Opt. Soc. Am..

[20] Benayas A., Silva W.F., Rodenas A., Jacinto C., Vázquez de Aldana J., Chen F., Tan Y., Thomson R.R., Psaila N.D., Reid D.T. (2011). Ultrafast laser writing of optical waveguides in ceramic Yb:YAG: A study of thermal and non-thermal regimes. Appl. Phys. A.

[21] Sola D., Escartin A., Cases R., Peña J.I. (2011). Crystal growth induced by Nd:YAG laser irradiation in patterning glass ceramic substrates with dots. Opt. Mater..

[22] Xu L., Knox W.H. (2011). Lateral gradient index microlenses written in ophthalmic hydrogel polymers by femtosecond laser machining. Opt. Mater. Express.

[23] Sola D., Martinez de Mendibil J., Vazquez de Aldana J.R., Lifante G., Balda R., de Aza A.H., Pena P., Fernandez J. (2013). Stress-induced buried waveguides in the 0.8CaSiO_3_-0.2Ca_3_(PO_4_)_2_ eutectic glass doped with Nd^3+^ ions. Appl. Surf. Sci..

[24] Chen F., Vazquez de Aldana J.R. (2014). Optical waveguides in crystalline dielectric materials produced by femtosecond-laser micromachining. Laser Photonics Rev..

[25] Martinez de Mendivil J., Sola D., Vazquez de Aldana J.R., Lifante G., de Aza A.H., Pena P., Peña J.I. (2015). Ultrafast direct laser writing of cladding waveguides in the 0.8CaSiO_3_-0.2Ca_3_(PO_4_)_2_ eutectic glass doped with Nd^3+^ ions. J. Appl. Phys..

[26] Srinivasan R., Mayne-Banton V. (1982). Self-developing photoetching of poly(ethylene terephthalate) films by far-ultraviolet excimer laser radiation. Appl. Phys. Lett..

[27] Kawamura Y., Toyoda K., Namba S. (1982). Effective deep ultraviolet photoetching of polymethyl methacrylate by an excimer laser. Appl. Phys. Lett..

[28] Srinivasan V., Smrtic M.A., Badu S.V. (1986). Excimer laser etching of polymers. J. Appl. Phys..

[29] Srinivasan R., Braren B., Casey K.G. (1990). Nature of incubation pulses in the ultraviolet laser ablation of polymethyl methacrylate. J. Appl. Phys..

[30] Cain S.R., Burns F.C., Otis C.E. (1992). On single-photon ultraviolet ablation of polymeric materials. J. Appl. Phys..

[31] Blanchet G.B., Cotts P., Fincher C.R. (2000). Incubation: Subthreshold ablation of poly-(methyl methacrylate) and the nature of the decomposition pathways. J. Appl. Phys..

[32] Schaffer C.B., Brodeur A., Garcia J.F., Mazur E. (2001). Micromachining bulk glass by use of femtosecond laser pulses with nanojoule energy. Opt. Lett..

[33] Schaffer C.B., Garcia J.F., Mazur E. (2003). Bulk heating of transparent materials using a high-repetition-rate femtosecond laser. Appl. Phys. A.

[34] Eaton S.M., Zhang H., Herman P.R., Yoshino F., Shah L., Bovatsek J., Arai A.Y. (2005). Heat accumulation effects in femtosecond laser-written waveguides with variable repetition rate. Opt. Express.

[35] Eaton S.M., Zhang H., Ng M.L., Li J., Chen W., Ho S., Herman P.R. (2008). Transition from thermal diffusion to heat accumulation in high repetition rate femtosecond laser writing of buried optical waveguides. Opt. Express.

[36] Sola D., Lavieja C., Orera A., Clemente M.J. (2018). Direct laser interference patterning of ophthalmic polydimethylsiloxane (PDMS) polymers. Opt. Lasers Eng..

[37] Sola D., Alamri S., Lasagni A.F., Artal P. (2019). Fabrication and characterization of diffraction gratings in ophthalmic polymers by using UV direct laser interference patterning. Appl. Surf. Sci..

[38] Huang R., Knox W.H. (2019). Femtosecond micro-machining of hydrogels: Parametric study and photochemical model including material saturation. Opt. Mater. Express.

[39] Campaign S.M.G., Knox W.H. (2019). Increase in efficacy of near-infrared femtosecond micromachining in ophthalmic hydrogels with the addition of sodium fluorescein, rose bengal, and riboflavin. Appl. Opt..

[40] Sola D., Cases R. (2020). High-repetition-rate femtosecond laser processing of acrylic intra-ocular lenses. Polymers.

[41] Abasi S., Podstawczyk D.A., Sherback A.F., Guiseppi-Elie A. (2019). Biotechnical properties of poly(HEMA-co-HPMA) hydrogels are governed by distribution among water states. ACS Biomater. Sci. Eng..

[42] Gamaly E.G., Rode A.V., Luther-Davies B. (1999). Ultrafast laser ablation with high-pulse-rate lasers. Part I: Theoretical considerations. J. Appl. Phys..

[43] Taddei P., Balducci F., Simoni R., Monti P. (2005). Raman, IR and thermal study of a new highly biocompatible phosphorylcholine-based contact lens. J. Mol. Struct..

[44] Bertoluzza A., Monti P., Garcia-Ramos J.V., Simoni R., Caramazza R., Calzavara A. (1986). Applications of Raman spectroscopy to the ophthalmological field: Raman spectra of soft contact lenses made of poly-2-hydroxyethylmethacrylate (PHEMA). J. Mol. Struct..

[45] Ding L., Cancado L.G., Novotny L., Knox W.H., Anderson N., Jani D., Linhardt J., Blackwell R.I., Künzler J.F. (2009). Micro-Raman spectroscopy of refractive index microstructures in silicone-based hydrogel polymers created by high-repetition-rate femtosecond laser micromachining. J. Opt. Soc. Am. B.

[46] Mailis S., Anderson A.A., Barrington S.J., Brocklesby W.S., Greef R., Rutt H.N., Eason R.W., Vainos N.A., Grivas G. (1998). Photosensitivity of lead germanate glass waveguides grown by pulsed laser deposition. Opt. Lett..

[47] Park J.K., Cho S.H. (2011). Flexible gratings fabricated in polymeric plate using femtosecond laser irradiation. Opt. Lasers Eng..

[48] Scully P.J., Jones D., Jaroszynski D.A. (2003). Femtosecond laser irradiation of polymethylmethacrylate for refractive index gratings. J. Opt. A Pure Appl. Opt..

[49] Kallepalli D.L.N., Desai N.R., Soma V.R. (2010). Fabrication and optical characterization of microstructures in poly(methylmethacrylate) and poly(dimethylsiloxane) using femto second pulses for photonic and microfluidic applications. Appl. Opt..

[50] Deepak K.L.N., Rao S.V., Rao D.N. (2010). Femtosecond laser-fabricated microstructures in bulk poly(methylmethacrylate) and poly(dimethylsiloxane) at 800 nm towards lab-on-a-chip applications. Pramana J. Phys..

